# Draft genome sequence of *Brucella canis* strain isolated from Mexico State, Mexico

**DOI:** 10.1128/mra.00458-25

**Published:** 2025-08-27

**Authors:** Christian Atayde-Torres, Stephanie Viridiana Laredo-Tiscareño, Rodolfo Gonzalez-Peña, Erika G. Palomares-Resendiz, Erick de Jesús de Luna-Santillana, Jaime R. Adame-Gallegos, Carlos A. Rodríguez-Alarcón, Nestor Fabián Galvis-Serrano, Javier A. Garza-Hernández

**Affiliations:** 1 Instituto de Ciencias Biomédicas, Universidad Autónoma de Ciudad Juárez 27764, Ciudad Juarez, Chihuahua, Mexico; 2 Centro de Investigaciones Regionales “Dr. Hideyo Noguchi”, Universidad Autónoma de Yucatán 27778, Mérida, Yucatan, Mexico; 3 CENID-SAI Instituto Nacional de Investigaciones Forestales, Cuajimalpa, Ciudad de México, Mexico; 4 Centro de Biotecnología Genómica del Instituto Politécnico Nacional, Reynosa, Tamaulipas, Mexico; 5 Universidad Autónoma de Chihuahua Facultad de Ciencias Químicas 528981 , Chihuahua, Mexico; 6 Facultad de Ciencias Medicas y de la Salud, Grupo de Investigación Biogen, Universidad de Santander, Cúcuta, Colombia.; Portland State University, Portland, Oregon, USA

**Keywords:** *Brucella canis*, genome analysis, Mexico

## Abstract

*Brucella canis* is a zoonotic Gram-negative coccobacillus of significant public health concern. This bacterium harbors multiple virulence factors that facilitate infection. We report the draft genome sequence of a *B. canis* strain isolated from an infected dog in Mexico and provide an annotation of genes associated with its virulence factors.

## ANNOUNCEMENT

*Brucella canis* is the etiological agent of canine brucellosis and a zoonotic pathogen capable of infecting humans (1).

In Mexico, *B. canis* is endemic in several states, causing abortions and severe illnesses in dogs. Additionally, evidence suggests that it has also infected humans (2, 3). On 10 February 2024, we isolated a *B. canis* strain from the vaginal fluid of an infected female dog that had previously experienced an abortion in the State of Mexico, Mexico (19°19′33″N, 99°10′16″W; 2,271 masl). The genetic composition of the bacterium was sequenced and analyzed to identify genes associated with virulence factors. Initially, the bacterium was isolated using tryptic soy agar (TSA) plates incubated for 10 days at 37°C. A colony was then re-isolated on fresh TSA, and Gram staining was performed to confirm the purity of the culture. The VITEK 2 automated microbiology system (bioMérieux, Inc., Hazelwood, MO, USA) was used for species confirmation. DNA was then extracted from colonies grown on fresh TSA medium using the Qiagen Blood and Tissue kit (Qiagen, Germany, Cat. No. 69506), following the manufacturer’s instructions.

Genomic DNA was sequenced following the protocol described by Alemán-Huerta et al. (4), using 500 ng of genomic DNA. Briefly, DNA quantification was performed using the Qubit double-stranded DNA high-sensitivity assay kit on a Qubit fluorometer (Thermo Fisher Scientific, MA, USA). Library preparation was conducted with the Nextera Flex library kit, incorporating individual indices for barcoding, following the Illumina reference protocol (10000000254). Finally, sequencing was carried out using Illumina MiniSeq technology.

A total of 1,534,506 paired-end 145 bp reads were obtained, with a sequencing depth of >300×, after a quality check using FastQC v0.11.9 in Galaxy v1.0.1 (5). Default parameters were used for all software unless otherwise specified. The raw reads were trimmed using Trimmomatic v0.39 (6) to remove adapters and improve sequence quality. The trimmed reads were then assembled using SPAdes v3.15.3 (7) on the Unicycler v0.5.1 (8) online platform. The assembled reads produced multiple contigs, which were subsequently reduced to 30 contigs using the RaqTaq v2.1.0 genome assembler (9) with a single-contig *B. canis* isolate as a reference (accession numbers NC_010103 and NC_010104 for chromosome 1 and chromosome 2, respectively). The final set of contigs was annotated using Prokka v1.14.6 (10), and the publicly available genome was annotated using the NCBI Prokaryotic Genome Annotation Pipeline (PGAP) (11). The genome comprised 3,268,571 bp in 32 contigs and is composed of two chromosomes that are 2,060,973 and 1,207,598 bp in length. The N_50_ value of 169,424 bp and a GC content of 57.24%. It contains 6,084 genes, including 3,070 coding sequences (CDS), 3 rRNA genes, 47 tRNA genes, and 1 tmRNA gene. Using the RAST annotation tool (12) and ABRicate tool for mass screening of contigs for antimicrobial and virulence genes in Galaxy server (13), 42 putative genes associated with virulence factors were identified in *B. canis*. Table 1 shows a comprehensive list of virulence-associated genes, including their genome sizes and genomic positions.

**TABLE 1:** Putative virulence genes in the Mexican *Brucella canis* strain identified using the virulence factors database (VFDB) as reference^*a*^

Gene	Start—End	Strand	% Coverage	% Identity	Accession	Product
lpsB/lpcC	125,473 — 126,537	+	100	99.62	NP_539426	Lipopolysaccharide core biosynthesis mannosyltransferase LpcC (LPS [CVF383]) (*Brucella melitensis* bv. 1 str. 16M)
lpxC	204,442 — 205,302	+	100	99.65	NP_539503	UDP-3-O-(3-hydroxymyristoyl) N-acetylglucosamine deacetylase (LPS [CVF383]) (*Brucella melitensis* bv. 1 str. 16M)
ricA	358,854 — 359,381	+	100	100	NP_539653	Rab2 interacting conserved protein A (RicA [VF0414]) (*Brucella melitensis* bv. 1 str. 16M)
lpxD	450,886 — 451,941	+	100	99.81	NP_539748	UDP-3-O-(3-hydroxymyristoyl) glucosamine N-acyltransferase (LPS [CVF383]) (*Brucella melitensis* bv. 1 str. 16M)
fabZ	451,886 — 452,407	+	100	99.62	NP_539749	(3R)-hydroxymyristoyl ACP dehydratase (LPS [CVF383]) (*Brucella melitensis* bv. 1 str. 16M)
lpxA	452,404 — 453,252	+	100	100	NP_539750	UDP-N-acetylglucosamine acyltransferase (LPS [CVF383]) (*Brucella melitensis* bv. 1 str. 16M)
lpxB	454,132 — 455,319	+	100	99.83	NP_539752	Lipid-A-disaccharide synthase (LPS [CVF383]) (*Brucella melitensis* bv. 1 str. 16M)
kdsA	472,176 — 473,009	+	100	99.88	NP_539767	2-dehydro-3-deoxyphosphooctonate aldolase (LPS [CVF383]) (*Brucella melitensis* bv. 1 str. 16M)
acpXL	185,874 — 186,110	+	100	100	NP_540392	Acyl carrier protein (LPS [CVF383]) (*Brucella melitensis* bv. 1 str. 16M)
wbpL	239,207 — 240,124	-	91.07	99.89	NP_540343	Undecaprenyl-phosphate alpha-N-acetylglucosaminyltransferase (LPS [CVF383]) (*Brucella melitensis* bv. 1 str. 16M)
kdsB	43,151 — 44,038	-	100	99.89	NP_540821	3-deoxy-manno-octulosonate cytidylyltransferase (LPS [CVF383]) (*Brucella melitensis* bv. 1 str. 16M)
virB10	43 — 864	+	100	97.08	NP_541011	Type IV secretion system channel protein VirB10 (VirB type IV secretion system [VF0365]) (*Brucella melitensis* bv. 1 str. 16M)
virB11	848 — 1,933	+	100	99.63	NP_541012	Type IV secretion system protein ATPase VirB11 (VirB type IV secretion system [VF0365]) (*Brucella melitensis* bv. 1 str. 16M)
virB12	2,079 — 2,597	+	100	99.23	NP_541013	Type IV secretion system protein VirB12 (VirB [SS043]) (*Brucella melitensis* bv. 1 str. 16M)
acpXL	62,439 — 62,720	+	100	100	NP_540028	Acyl carrier protein (LPS [CVF383]) (*Brucella melitensis* bv. 1 str. 16M)
htrB	66,455 — 67,378	+	100	99.67	NP_540032	Lipid A biosynthesis lauroyl acyltransferase (LPS [CVF383]) (*Brucella melitensis* bv. 1 str. 16M)
wboA	2,921 — 4,153	+	100	99.76	NP_539915	Glycosyltransferase (LPS [CVF383]) (*Brucella melitensis* bv. 1 str. 16M)
wbdA	4,150 — 5,709	+	99.94	99.42	NP_539914	Mannosyltransferase (LPS [CVF383]) (*Brucella melitensis* bv. 1 str. 16M)
btpB	87,915 — 88,748	+	100	100	NP_697749	Tir domain containing protein BtpB (BtpB [VF0522)] (*Brucella suis* 1330)
lpxE	91,737 — 92,444	+	100	99.72	NP_540129	Phosphatidylglycerophosphatase B (LPS [CVF383]) (*Brucella melitensis* bv. 1 str. p6M)
pmm	793 — 2,168	+	100	99.42	NP_540313	Phosphomannomutase (LPS [VF0367]) (*Brucella melitensis* bv. 1 str. 16M)
manCoAg	2,239 — 3,663	+	100	99.86	NP_540312	Mannose-1-phosphate guanylyltransferase (LPS [VF0367]) (*Brucella melitensis* bv. 1 str. 16M)
manAoAg	3,705 — 4,868	+	100	99.91	NP_540311	Mannose-6-phosphate isomerase (LPS [VF0367]) (*Brucella melitensis* bv. 1 str. 16M)
wbpZ	4,927 — 6,060	+	100	99.82	NP_540310	Mannosyltransferase C (LPS [CVF383]) (*Brucella melitensis* bv. 1 str. 16M)
lpsA	78,152 — 80,317	-	100	99.77	NP_540243	Glycosyltransferase (LPS [CVF383]) (*Brucella melitensis* bv. 1 str. 16M)
cgs	27,121 — 35,724	+	99.98	99.7	NP_540754	Cyclic beta 1-2 glucan synthetase (C < beta > G [VF0366]) (*Brucella melitensis* bv. 1 str. 16M)
pgm	22 — 1,722	-	100	99.82	NP_540803	Phosphoglucomutase (LPS [VF0367]) (*Brucella melitensis* bv. 1 str. 16M)
virB1	5,731 — 6,447	+	100	99.86	NP_540803	Type IV secretion system attachment mediating protein VirB1 Homolog (VirB Type IV secretion system [VF0365]) (*Brucella melitensis* bv. 1 str. 16M)
virB2	6,883 — 7,200	+	100	99.37	NP_541003	Type IV secretion system protein VirB2 (VirB Type IV secretion system [VF0365]) (*Brucella melitensis* bv. 1 str. 16M)
virB3	7,214 — 7,562	+	99.43	100	NP_541004	Type IV secretion system protein VirB3 (VirB Type IV secretion system [VF0365]) (*Brucella melitensis* bv. 1 str. 16M)
virB4	7,564 — 10,059	+	100	99.68	NP_541005	Type IV secretion system protein ATPase VirB4 (VirB Type IV secretion system [VF0365]) (*Brucella melitensis* bv. 1 str. 16M)
virB5	10,064 — 10,780	+	100	99.86	NP_541006	Type IV secretion system protein VirB5 (VirB Type IV secretion system [VF0365]) (*Brucella melitensis* bv. 1 str. 16M)
virB6	10,964 — 12,007	+	100	99.71	NP_541006	Type IV secretion system channel protein VirB6 (VirB Type IV secretion system [VF0365]) (*Brucella melitensis* bv. 1 str. 16M)
virB7	12,171 — 12,344	+	100	100	NP_541008	Type IV secretion system protein VirB7 (VirB Type IV secretion system [VF0365]) (*Brucella melitensis* bv. 1 str. 16M)
virB8	12,347 — 13,066	+	100	99.86	NP_541009	Type IV secretion system protein VirB8 (VirB Type IV secretion system [VF0365]) (*Brucella melitensis* bv. 1 str. 16M)
virB9	13,189 — 13,932	+	100	99.73	NP_541010	Type IV secretion system channel protein VirB9 (VirB Type IV secretion system [VF0365]) (*Brucella melitensis* bv. 1 str. 16M)
gmd	511 — 1,622	+	100	97.84	NP_540330	GDP-mannose 46-dehydratase (LPS [VF0367]) (*Brucella melitensis* bv. 1 str. 16M)
per	1,591 — 2,733	+	100	99.83	NP_540331	Perosamine synthetase (LPS [VF0367]) (*Brucella melitensis* bv. 1 str. 16M)
wzm	2,733 — 3,530	+	100	100	NP_540332	O-antigen export system permease protein (LPS [VF0367]) (*Brucella melitensis* bv. 1 str. 16M)
wzt	3,527 — 4,285	+	100	100	NP_540333	O-antigen export system ATP-binding protein (LPS [VF0367]) (*Brucella melitensis* bv. 1 str. 16M)
wbkB	4,282 — 5,136	+	100	99.65	NP_540334	Perosamine synthetase WbkB (LPS [VF0367]) (*Brucella melitensis* bv. 1 str. 16M)
wbkC	5,163 — 5,942	+	100	100	NP_540335	GDP-mannose 46-dehydratase / GDP-4-amino-46-dideoxy-D-mannose formyltransferase (LPS [VF0367]) (*Brucella melitensis* bv. 1 str. 16M)		

^*a*^ Strand (+): coding or sense strand. Strand (-):noncoding or antisense strand.

## Data Availability

The draft genome sequence of *B. canis* was deposited at GenBank under accession number JBMPGM000000000. The project data are available under BioProject acc. PRJNA1243460 and BioSample acc. SAMN47622929. The raw draft genome data were deposited in the Sequence Read Archive (SRA) under SRA acc. SRR33291349. Ethical approval was not required for this study.

## References

[1] Santos RL, Souza TD, Mol JPS, Eckstein C, Paíxão TA. 2021. Canine brucellosis: an update. Front Vet Sci 8:594291. doi:10.3389/fvets.2021.594291 33738302 PMC7962550

[2] Morales-Garcia M-G, Lopez-Mendez J, Pless R, Garcia-Morales E, Kosanke H, Hernandez-Castro R, Bedi J, Lopez-Merino A, Velazquez-Guadarram N, Jimenez-Rojas L, Rontreras-Rodriguez A. 2015. Brucellosis outbreak in a rural endemic region of Mexico - a comprehensive investigation. Vet Ital 51:185–190. doi:10.12834/vetit.305.3393.1 26455370

[3] Arciga-Vázquez GS, Santos-López G, Castañeda-Roldán EI, Cedillo-Ramírez ML, Cano-Vázquez EN, Monroy-Azuara MG, López-Méndez AI, Ayón-Aguilar J, Méndez-Martínez S. 2021. Estudio de casos confirmados de brucelosis humana en Puebla, México. Rev chil infectol 38:281–289. doi:10.4067/S0716-10182021000200281 34184720

[4] Alemán-Huerta ME, Martínez-Herrera RE, Elufisan TO, Gandarilla-Pacheco FL, Quintero-Zapata I, Reyez-López MÁ, de Luna-Santillana E de J. 2022. Draft genome sequence of a polyhydroxyalkanoate-producing Bacillus cereus strain isolated from Nuevo Leon State, Mexico. Microbiol Resour Announc 11:e0026922. doi:10.1128/mra.00269-22 35652668 PMC9302066

[5] Andrews S. 2010. FastQC: a quality control tool for high throughput sequence data. Available from: http://www.bioinformatics.babraham.ac.uk/projects/fastqc

[6] Bolger AM, Lohse M, Usadel B. 2014. Trimmomatic: a flexible trimmer for Illumina sequence data. Bioinformatics 30:2114–2120. doi:10.1093/bioinformatics/btu170 24695404 PMC4103590

[7] Prjibelski A, Antipov D, Meleshko D, Lapidus A, Korobeynikov A. 2020. Using SPAdes de novo assembler. CP in Bioinformatics 70:e102. doi:10.1002/cpbi.102 32559359

[8] Wick RR, Judd LM, Gorrie CL, Holt KE. 2017. Unicycler: resolving bacterial genome assemblies from short and long sequencing reads. PLoS Comput Biol 13:e1005595. doi:10.1371/journal.pcbi.1005595 28594827 PMC5481147

[9] Alonge M, Lebeigle L, Kirsche M, Jenike K, Ou S, Aganezov S, Wang X, Lippman ZB, Schatz MC, Soyk S. 2022. Automated assembly scaffolding using RagTag elevates a new tomato system for high-throughput genome editing. Genome Biol 23:258. doi:10.1186/s13059-022-02823-7 36522651 PMC9753292

[10] Seemann T. 2014. Prokka: rapid prokaryotic genome annotation. Bioinformatics 30:2068–2069. doi:10.1093/bioinformatics/btu153 24642063

[11] Tatusova T, DiCuccio M, Badretdin A, Chetvernin V, Nawrocki EP, Zaslavsky L, Lomsadze A, Pruitt KD, Borodovsky M, Ostell J. 2016. NCBI prokaryotic genome annotation pipeline. Nucleic Acids Res 44:6614–6624. doi:10.1093/nar/gkw569 27342282 PMC5001611

[12] Aziz RK, Bartels D, Best AA, DeJongh M, Disz T, Edwards RA, Formsma K, Gerdes S, Glass EM, Kubal M, et al.. 2008. The RAST Server: rapid annotations using subsystems technology. BMC Genomics 9:75. doi:10.1186/1471-2164-9-75 18261238 PMC2265698

[13] Seemann T. 2016. ABRicate: mass screening of contigs for antibiotic resistance genes. GitHub, San Francisco, CA. Available from: https://github.com/tseemann/abricate

